# PUFchain 3.0: Hardware-Assisted Distributed Ledger for Robust Authentication in Healthcare Cyber–Physical Systems

**DOI:** 10.3390/s24030938

**Published:** 2024-01-31

**Authors:** Venkata K. V. V. Bathalapalli, Saraju P. Mohanty, Elias Kougianos, Vasanth Iyer, Bibhudutta Rout

**Affiliations:** 1 Department of Computer Science and Engineering, University of North Texas, Denton, TX 76203, USA; vb0194@unt.edu; 2 Department of Electrical Engineering, University of North Texas, Denton, TX 76203, USA; 3 Department of Computer Science and Digital Technologies, Grambling State University, Grambling, LA 71245, USA; iyerv@gram.edu; 4 Department of Physics, University of North Texas, Denton, TX 76201, USA; bibhudutta.rout@unt.edu

**Keywords:** smart healthcare, healthcare cyber–physical systems (H-CPS), physical unclonable function (PUF), hardware-assisted security (HAS), masked authentication messaging (MAM), security-by-design (SbD), blockchain, Tangle

## Abstract

This article presents a novel hardware-assisted distributed ledger-based solution for simultaneous device and data security in smart healthcare. This article presents a novel architecture that integrates PUF, blockchain, and Tangle for Security-by-Design (SbD) of healthcare cyber–physical systems (H-CPSs). Healthcare systems around the world have undergone massive technological transformation and have seen growing adoption with the advancement of Internet-of-Medical Things (IoMT). The technological transformation of healthcare systems to telemedicine, e-health, connected health, and remote health is being made possible with the sophisticated integration of IoMT with machine learning, big data, artificial intelligence (AI), and other technologies. As healthcare systems are becoming more accessible and advanced, security and privacy have become pivotal for the smooth integration and functioning of various systems in H-CPSs. In this work, we present a novel approach that integrates PUF with IOTA Tangle and blockchain and works by storing the PUF keys of a patient’s Body Area Network (BAN) inside blockchain to access, store, and share globally. Each patient has a network of smart wearables and a gateway to obtain the physiological sensor data securely. To facilitate communication among various stakeholders in healthcare systems, IOTA Tangle’s Masked Authentication Messaging (MAM) communication protocol has been used, which securely enables patients to communicate, share, and store data on Tangle. The MAM channel works in the restricted mode in the proposed architecture, which can be accessed using the patient’s gateway PUF key. Furthermore, the successful verification of PUF enables patients to securely send and share physiological sensor data from various wearable and implantable medical devices embedded with PUF. Finally, healthcare system entities like physicians, hospital admin networks, and remote monitoring systems can securely establish communication with patients using MAM and retrieve the patient’s BAN PUF keys from the blockchain securely. Our experimental analysis shows that the proposed approach successfully integrates three security primitives, PUF, blockchain, and Tangle, providing decentralized access control and security in H-CPS with minimal energy requirements, data storage, and response time.

## 1. Introduction

**Figure 1:**
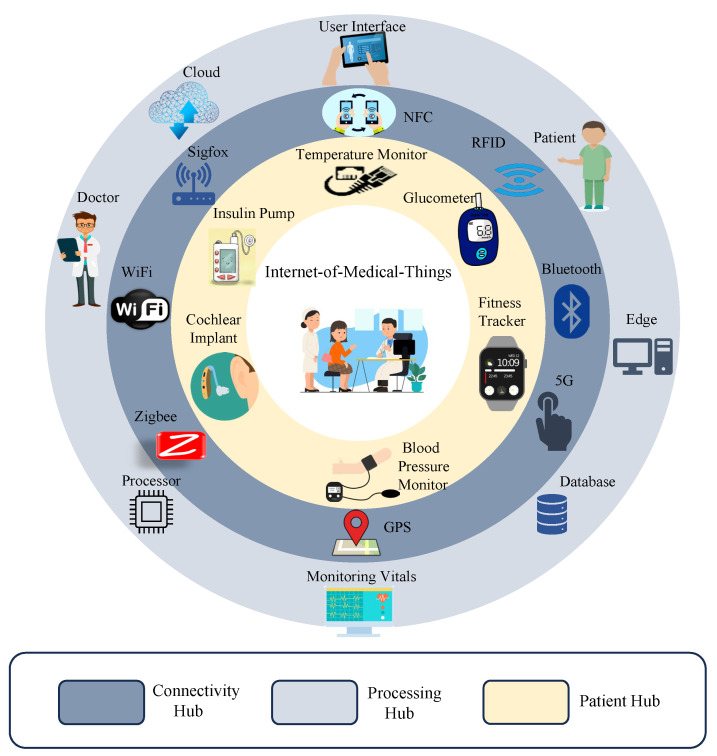
Healthcare cyber–physical system.

The application of IoMT has made healthcare systems more advanced by integrating various technologies like machine learning (ML), big data, and blockchain [1,2]. Smart e-health service applications are becoming more adaptable through the integration of Medtronic devices for patient physiological metrics monitoring and sensing. Telemedicine, e-health, and connected health are emerging healthcare ecosystems with advanced network communication technologies like 5G, and 6G supporting data sensing, communication, and analysis through AI and ML technologies. Medtronic devices play an important role in realizing the potential of these applications. However, the potential security vulnerabilities have made device integrity, data confidentiality, and privacy pivotal for H-CPS [2]. The architectural overview of healthcare cyber–physical systems is presented in Figure 1.

### Cybersecurity in Smart Healthcare

**Figure 2:**
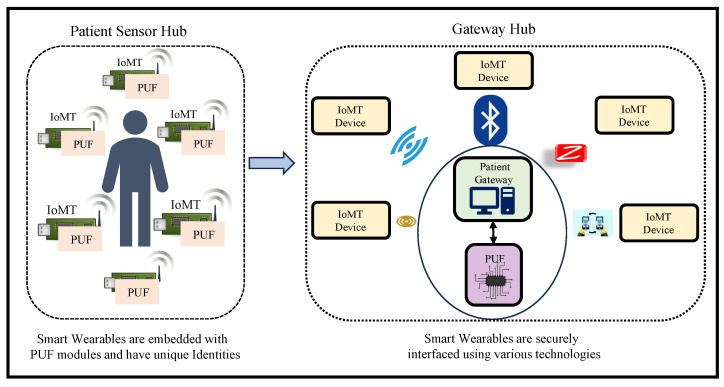
Patient’s Body Area Network.

IoMT is a collection of heterogeneous smart Medtronic devices with diverse functionalities and capabilities that can sense and process various parameters and are grouped as a hub on the patient to analyze the patient’s physiological parametric data as shown in Figure 2. The data from these heterogeneous devices are analyzed and processed for effective analysis, decision making, and monitoring of patient health [3,4]. These devices are not computationally capable of processing the data and require ML- and AI-supported capabilities for processing and decision making, which can be supported by edge, cloud, and fog computing paradigms. Wearable and implantable medical electronic devices are placed inside and, on the body, to monitor various physiological parameters and generate data. These devices can be smart pumps to deliver insulin dosage, pacemakers that can simulate neurological signals inside the brain, and an active fitness tracker monitoring heart rate and blood pressure [5,6]. Various security attacks are possible through eavesdropping, spoofing, and sniffing to obtain sensitive patients’ physiological information using security vulnerabilities associated with the system. An adversary can intercept the communication between an IoMT device and the health service entity with computing capabilities to obtain access to the system and control it. This can pose a question regarding data integrity and device authenticity in IoMT, which may jeopardize healthcare service applications [7,8].

To address the data privacy issues in smart healthcare, many researchers have adopted distributed ledger technology (DLT)-based solutions that provide immutability and confidentiality to data [9,10]. DLT can facilitate authorized access to data and can counter any adversarial measure to tamper with the data. These functionalities have made the DLT-based approach for providing security and privacy to data more alluring, specifically in the areas of banking, finance, e-health, and smart cities which demand the utmost secrecy and confidentiality of data in their applications.

IoMT devices are vulnerable to various types of physical attacks [11,12]. Cybersecurity solutions are often based on software-based approaches that work based on symmetric and asymmetric key cryptography schemes. These approaches require non-volatile memory or drives for key storage and retrieval. Using asymmetric keys for encryption and data decryption can sometimes restrict access to medical professionals or patients [13]. This sort of dependence on memory has made these security protocols more vulnerable to various ML attacks, where an attacker can obtain access to the secret key and the system [14]. SbD is one of the new paradigms that has attracted much attention from the research community. This approach focuses on building a security model right from the design stage. PUF is a prominent SbD that is a unique hardware identity generation scheme.

Various hardware-assisted security (HAS) approaches for cybersecurity are being adopted using PUF and Trusted Platform Modules (TPMs) to achieve the objective of SbD [11,15]. PUF-based security solutions include a PUF module that is embedded in a chip and can generate keys from the PUF design using process variations inside an Integrated Circuit (IC) [11,15,16]. The generated keys can be used as security keys or identities for that PUF module on the chip. PUFs do not require a database for key storage, and PUF responses are generated instantly by taking advantage of micro-manufacturing process variations during chip fabrication [15,17,18]. Data confidentiality, integrity, privacy, and device authentication are requirements for sustainable SC. Blockchain has been one of the most widely explored DLTs for financial transactions since its inception in 2008 [19]. However, resource-constrained IoT devices cannot sustain the computational resource requirements of blockchain’s consensus mechanisms like Proof-of-Work (PoW). Data immutability, integrity, and privacy in SC are guaranteed by blockchain through its scalable, decentralized physiological data management using energy-efficient consensus mechanisms [11].

**Figure 3:**
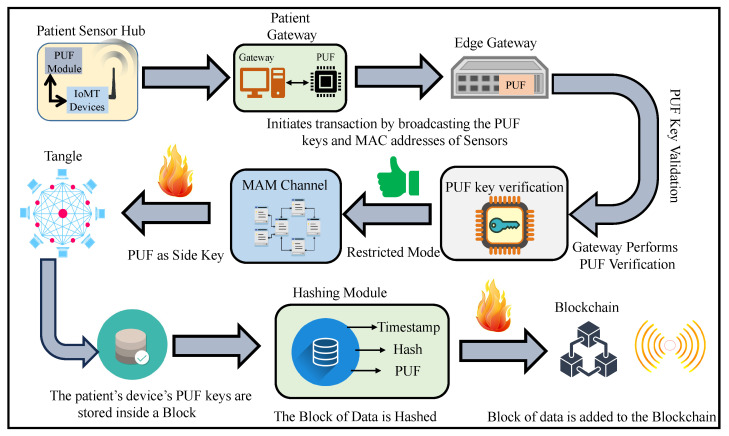
Architectural overview of proposed SbD approach for H-CPS.

The motivation for this research is to ensure the security of IoMT devices and their data, where the patient’s BAN PUF keys are securely stored inside a global blockchain to provide end-point security. Tangle is used for the secure communication of the patient’s physiological sensor data, and their access is controlled using a unique identity generated by PUF for the patient’s gateway. The proposed architecture works on integrating PUF with a DLT for providing a sustainable security primitive for IoMT-driven smart healthcare as illustrated in Figure 3.

Following the introduction, the rest of the paper is organized as follows: Section 2 presents the novel contributions of this paper. Section 3 discusses various hardware security schemes and DLT-based solutions in SC from the literature. The conceptual overview of SbD and the role of PUF as a formidable security primitive is given in Section 4. Section 5 explains IOTA Tangle, transaction validation, and Masked Authentication Messaging (MAM) concepts. A brief overview of blockchain technology is given in Section 6. The working flow of the device authentication and transaction validation process in the proposed PUFchain 3.0 is explained in Section 7. Section 8 outlines the implementation details, and Section 9 presents the conclusion and directions for future research.

## 2. Novel Contributions

In this section, we explain the challenges and contributions of the present work in Section 2.1. We present the novelty and significance of our present work PUFchain 3.0 in Section 2.2. Finally, a brief overview of our PUFchain idea, first ever hardware-assisted blockchain, and its variants are given in Section 2.3.

### 2.1. Research Problems Addressed in the Current Paper

The proposed work was envisioned to address the following questions:

- To the best of our knowledge, very few security primitives work on providing device and data-assisted security simultaneously for e-Health applications;
- Security gaps associated with device integrity, data confidentiality, and authenticity in edge computing-driven H-CPS;
- Lack of scalable and energy-efficient security approach for resource-constrained distributed systems in H-CPS;
- Sustainable approach to the device integrity-based access control mechanism for electronic health records (EHRs) management;
- Energy-efficient PUF architectures that are effective against machine learning and other attacks;
- Lack of sustainable and energy-efficient hardware-assisted access control mechanisms to the distributed ledger;
- A secure communication interface between various stakeholders in H-CPS with defined access and security;
- Presenting a security framework that could be integrated into real-world healthcare applications;
- Providing a cost-effective innovative approach to integrate various technologies for cybersecurity in smart healthcare;
- Enabling a patient to embed smart health devices that are secure and non-vulnerable to security attacks.

### 2.2. Novel Contributions of This Article

**Figure 4:**
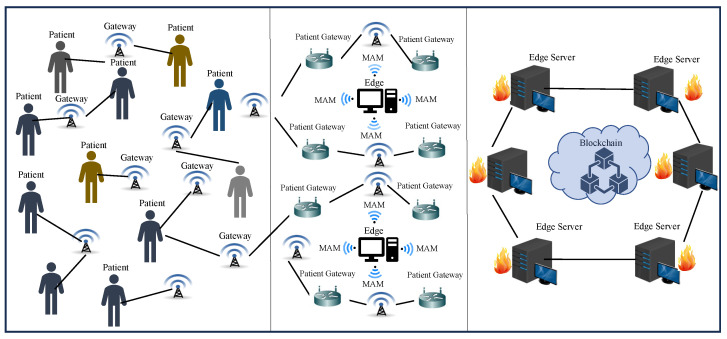
Layered view of PUFchain 3.0 Architecture.

- Presenting a novel state-of-the-art integration of PUF, blockchain, and Tangle for SbD of H-CPS. To the best of our knowledge, this is the first work on hardware-assisted security in H-CPS that presents a PUF-based approach for access to DLT for device and data security in H-CPS.
- Presenting a novel PUF-based access control mechanism for Tangle.
- A novel blockchain-integrated framework for security in H-CPS using smart contracts.
- Validating the proposed framework in the MAM “Restricted mode” for secure access control to Tangle using PUF.
- An energy-efficient SbD approach that uses delay Arbiter and XOR PUF architectures.
- An edge–cloud-driven approach for resource-constrained systems in H-CPS that has three layers—physical layer, edge layer, and blockchain layer as illustrated in Figure 4.
- A novel energy-efficient approach that works on blockchain using smart contracts for storing and retrieving PUF keys of IoMT devices inside a patient’s Body Area Network (BAN).
- A security approach that facilitates secure access to patients’ BAN and ensures the integrity of data from IoMT in resource-constrained distributed systems.

### 2.3. A Comprehensive Evaluation of PUFchain Primitives

**Figure 5:**
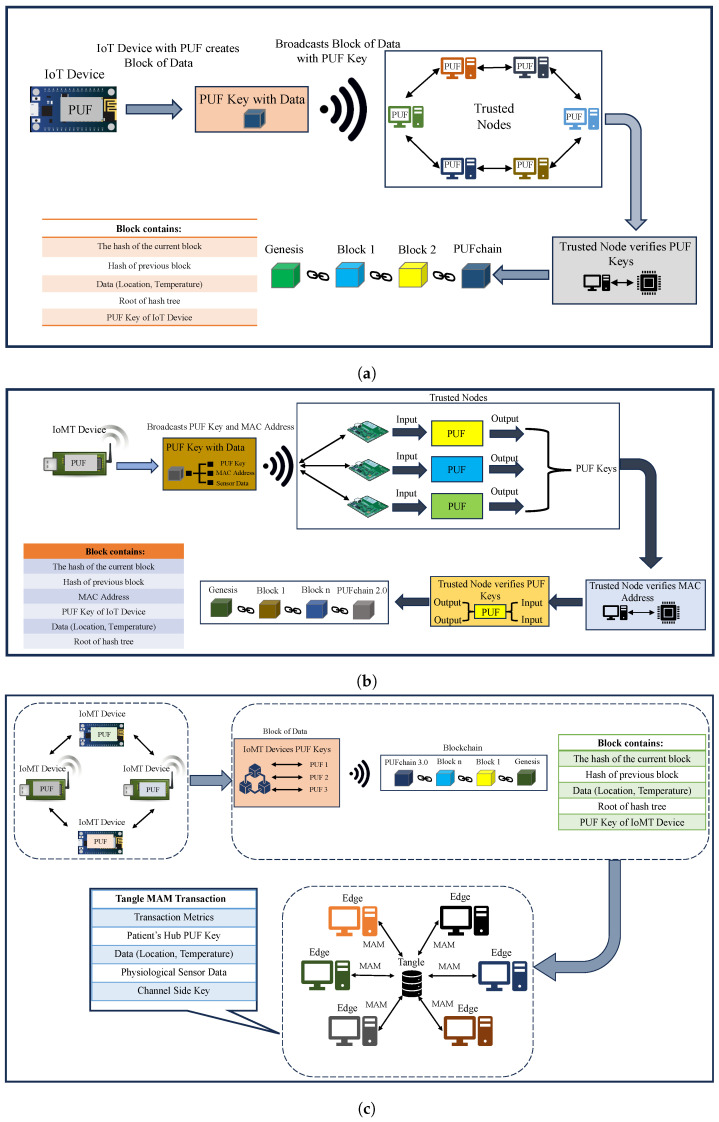
PUFchain variants. (**a**) PUFchain; (**b**) PUFchain 2.0; (**c**) proposed PUFchain 3.0.

The conceptual idea of PUFchain is presenting a hardware-assisted secure distributed ledger for sustainable device and data security in the emerging Internet of Everything (IoE). Hardware-assisted security involves embedding advanced electronic systems with PUF for device integrity. PUF-embedded security facilitates each electronic system to obtain a unique device identity that can relate to blockchain and other distributed ledgers. Table 1 and Figure 5 present a comparative analysis of our PUFchain variants.

## 3. Related Works

In this section, we have presented a brief review of related research on various distributed ledger technology-based cybersecurity solutions in smart healthcare. A comparative analysis of the proposed work PUFchain 3.0 with state-of-the-art research is given in Table 2.

Integration of hardware-assisted distributed ledger for SbD of CPS has gained prominence for addressing security gaps in various CPS, which include Healthcare CPS, Agriculture CPS, and Transportation CPS. The authors in [21] presented a scalable blockchain integrated distributed ledger solution for IoT applications. Their architecture has a blockchain running in the backend and a Tangle in the frontend. This approach claims to speed up the data processing from IoT devices by securely integrating with Tangle, which then offloads the data storage to blockchain in the cloud. In [22], the authors proposed an IC supply chain management system using PUF-based blockchain. Their work proposes a PUF-based chip-tracking system that uses blockchain to securely record and trace the ownership of a chip. A consensus mechanism for IoT applications is proposed in [23]. Their work presented a consensus mechanism titled “PoQDB”, which integrates blockchain with the CoBweb ledger to facilitate IoT data storage. The proposed work PUFchain 3.0 is an extension of the initially presented PUFchain [19], which is a novel integration of PUF and blockchain using a Proof-of-PUF-Enabled-Authentication (PoP) consensus mechanism for IoT security.

SbD of H-CPS is a focus area for many researchers since privacy and security issues have direct implications on the patient’s life. A smart, remote patient-monitoring system using IOTA is presented in [24]. The research proposed and validated an IOTA MAM-based approach for patient data access control and security. Using IPFS and MAM, their research validated an approach for patients’ IoT device control and access using a secure web interface. A blockchain-assisted solution for IoMT device security and access control is proposed in [25]. The motivation of their work is to provide security between different entities in healthcare systems. The blockchain-assisted IoMT key exchange mechanism is presented in [26]. Their work aims to address the single-point failure problem in processing data securely from IoMT devices. They presented a private consortium blockchain to validate the work and proposed a scheme for securely establishing communication between authenticated IoMT devices. However, their work uses cryptography to secure the keys of IoMT devices, which can be vulnerable to ML attacks. The authors in [9] proposed a secure IoMT data sharing scheme using IOTA MAM. Different modes of MAM were used to publish data onto Tangle, which included sensor and patient data. A PUF-based approach for the security of low-cost IoT devices in healthcare is proposed in [27], which presents a microcontroller based PUF that has 99% accuracy. The authors in [28] designed a blockchain-enabled IoMT device authentication architecture that presents an approach for encrypted communication and certificate-based identity attestation in IoMT. The detection of IoMT device malfunctioning and behavior is another efficient approach for device security. The authors in [29] presented a privacy-preserving IoMT device behavior detection using blockchain. In the paper, they validated this approach for insulin pumps to monitor patient’s glucose levels.

For sustainable device and data security in smart healthcare, we proposed a PUF-based blockchain solution named PUFchain 2.0 [11]. In this work, we validated and presented a PUF-based blockchain consensus mechanism for simultaneous device and data security. We observed the potential of hardware-assisted distributed ledgers for security in smart healthcare. The proposed PUFchain 3.0 work extends the potential of the PUF-based distributed ledger in smart healthcare by facilitating decentralized security and access control to IoMT devices and their data in H-CPS. In comparison with the related research, our work presents an architecture to address both device and data security with minimal latency and better scalability, thereby facilitating secure access control and security in smart healthcare.

**Table 2:** Comparative analysis with state-of-the-art research.

Research Works	Application	Security Primitive	Platform	Mechanism
Hellani et al., 2021 [21]	IoT (Data)	Blockchain and Tangle	Edge–Cloud	Smart Contracts
Mohanty et al., 2019 [19]	IoT (Device and Data)	PUF, blockchain	Edge	Proof-of-PUF-Enabled-Authentication
Al-Joboury et al., 2021 [23]	IoT (Data)	Blockchain and Cobweb	Cloud	IoT M2M Messaging (MQTT)
Wang et al., 2022 [30]	IoMT (Device)	Blockchain	Edge	Smart Contracts
Chaudhary et al., 2021 [22]	Hardware Supply Chain	PUF, blockchain	Edge–Cloud	Smart Contracts
Venkata et al., 2022 [11]	IoMT (Device)	PUF, blockchain	Edge	Media Access Control (MAC) and PUF-based Authentication
Satra et al., 2023 [14]	IoMT (Device)	PUF	Edge	Machine Learning
Fotopoulos et al., 2020 [28]	IoMT (Device)	Blockchain	-	Self- Sovereign Identity (SSI)
Zheng et al., 2023 [9]	IoMT (Data)	IOTA Tangle and blockchain	Edge	MAM
Proposed PUFchain 3.0 [20]	IoMT (Device and Data)	PUF, Tangle, blockchain	Edge–Cloud	Masked Authentication Messaging, smart contracts

## 4. Role of Physical Unclonable Functions as SbD Primitive

### 4.1. Security-by-Design

**Figure 6:**
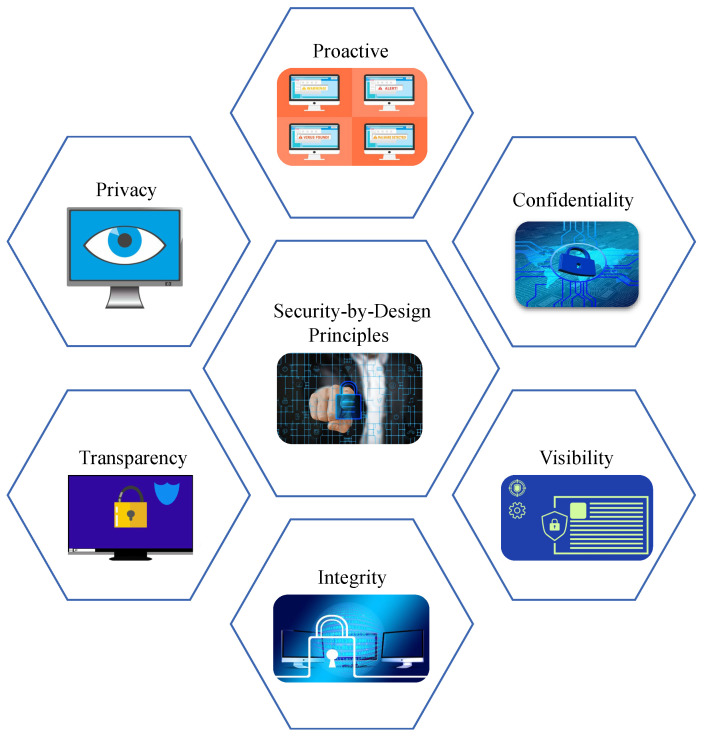
Security-by-Design principles.

SbD, or Privacy-by-Design (PbD), is a system development paradigm for smart electronics that emphasizes the security of an electronic system at the development stage, considering the intrinsic properties at the design, manufacturing, testing, and implementation. The principles and objectives of SbD as explained in Figure 6 mainly envision to avoid performance trade-offs in security primitives at the application stage of an electronic system [31,32].

The principles of SbD are as follows:

1. *Proactive but not reactive:* Existing cybersecurity solutions for smart electronics mostly focus on the security at application level. SbD promotes security as a design stage metric that is enabled by default.
2. *End-to-end security*: The security of the system should be considered right from the design stage to manufacturing, deployment, application, and maintenance.
3. *Security as default*: The security primitive should be enabled by default in the system and cannot be an optional primitive for the users to choose from.
4. *Least privilege*: Users of an electronic system should have the privilege of running the applications and should not have access to tamper with the system’s security specifications.
5. *Transparency*: The security principles should be clearly transparent and easily understandable. The users of an SbD-enabled system should have access to change their security level based on their choice and should be able to clearly understand its functionality.
6. *User centricity*: The ease of security principles and deployment is an essential aspect of SbD. The security primitives should not be burdensome for the users.
7. *Full functionality*: The security primitive should have efficient performance and should not have performance trade-offs that might impact the system’s functionality and applications.

### 4.2. PUF for SbD of H-CPS

**Figure 7:**
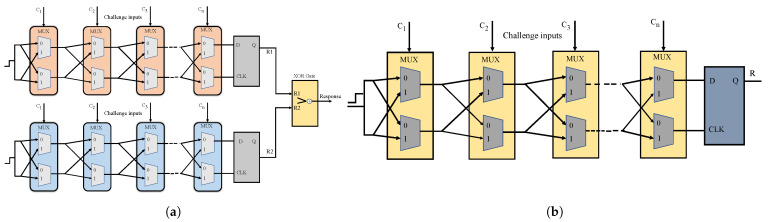
Architectures of delay PUFs experimentally validated in the proposed work. (**a**) XOR Arbiter PUF; (**b**) Arbiter PUF instance generating 1-bit output.

PUF is a hardware security primitive that uses device inherent manufacturing imperfections and generates a unique cryptographic identity. Each electronic device has a unique topology due to the manufacturing variations during the fabrication of an Integrated Circuit (IC), which is the building block of a consumer electronic system [33]. As each device has a distinct topology, unique keys can be derived based on its device property variations, such as frequency, delay, or the startup phase of a volatile memory cell. Process variations can be observed during various stages of an IC fabrication process, such as lithography, ion implantation, metallization, and packaging [20]. The variations introduced during these processes will slightly differentiate each device from the corresponding ones, even if they have the same fab, processes, and design. PUF works by deriving a key of random zeros and ones using the device’s intrinsic properties. PUFs can be classified based on the mapping of physical properties. PUF modules that work based on the propagation delays and frequency variations in an IC to build a unique bit stream are delay-based PUFs. Arbiter and Ring oscillator, XOR, and Butterfly PUF are widely used delay PUFs. These are also referred to as strong PUFs that can support the extraction of many random zeros and ones as a bit stream, which is essential for security applications. Similarly, Static Random Access Memory (SRAM) and Dynamic Random Access Memory (DRAM) are prominent memory PUF modules that work by generating a unique response based on the variations in the memory structures such as Flip Flops and an SRAM cell. The structure of Arbiter and XOR PUF used for experimental validation in this work is presented in Figure 7. The PUF module works on the physical randomness of devices by mapping a challenge input to a unique response output string. The uniqueness of this primitive is that it does not generate the same responses for varying challenge inputs. Also, two different PUF modules tested against the same challenge input will have varying bits of random zeros and ones as responses [19,34]. The responses from PUF are evaluated against various metrics to verify the strength of the keys. Some of the figures-of-merit (FoMs) of PUF are illustrated as follows:

- ***Uniqueness***: Verifying the extent of variation of the responses from a PUF circuit on two devices is referred to as uniqueness. This is measured by calculating the average inter-Hamming distances of responses from the PUF module on two devices tested with the same set of challenges.
- ***Reliability***: The stability of a PUF is determined by determining the variation of the responses under different environmental conditions. This is an essential metric in evaluating a PUF strength since the responses of the PUF must be stable under noise as well as under varying operating conditions.
- ***Randomness***: The randomness of a PUF is its ability to produce a response key with an equal number of randomly distributed ones and zeros. Ideally, a PUF response should have exactly an equal number of ones and zeros in the response bit stream.
- **Diffuseness:** The diffuseness of a PUF is obtained by calculating the average intra-Hamming distance of PUF responses to verify the extent of variation of responses for varying challenge inputs in the same PUF.

## 5. IOTA Tangle: A DAG Blockchain

IOTA Tangle is a DAG-based blockchain that has a Tangle structure. It is a distributed ledger from IOTA and one of the most suitable DLT-based solutions in IoT applications due to its miner and feeless functionality. All the transactions in Tangle are part of the directed acyclic graph (DAG) structure. The major advantage of this structure is that it increases the transaction validation rate exponentially when compared with the traditional blockchain structure that has all the transactions aligned sequentially [24]. Every new transaction on Tangle from a node validates the unconfirmed transactions called “Tips” to become part of the structure. Every incoming transaction validates tips using Proof-of-Work, and therefore increasing the number of incoming transactions substantially increases the rate of validated transactions. Tips are selected using the ‘Markov Chain Monte Carlo (MCMC)’ random walk algorithm which traverses the DAG and determines the transactions to be validated [35,36]. Proof-of-Work (PoW) validates a transaction by calculating the nonce and solving cryptographic puzzles. Once the tips are validated by an incoming transaction, then these transactions become confirmed in Tangle. PoW in Tangle is computationally resource efficient in comparison with blockchain’s PoW consensus mechanism [37].

Each transaction node in Tangle has a cumulative weight, which is calculated by adding its initial weight, and the cumulative weight of all the transactions directly or indirectly approve it [38,39]. In this DLT, a coordinator is responsible for overall transaction validation and approval. At present, the IOTA foundation is the coordinator that releases the milestones defining transaction validation rules. Simply, a coordinator is responsible for the overall functioning of the transaction validation approval process in Tangle [40]. A milestone is a stage where confirmed transactions become irreversible and final on Tangle [41].

IOTA MAM is a secure messaging protocol that operates on the IOTA main network for sending and receiving the encrypted information in Tangle through a channel by signing the message using the Merkle Hash Tree (MHT) signature algorithm. The message can be accessed by the receiver using the channel’s address, and whenever a new message of any length and size is uploaded on Tangle, a channel is created, and the receivers can immediately access the data using the root of the MHT. MAM operates in three different communication modes: public, private, and restricted [24].

**Figure 8:**
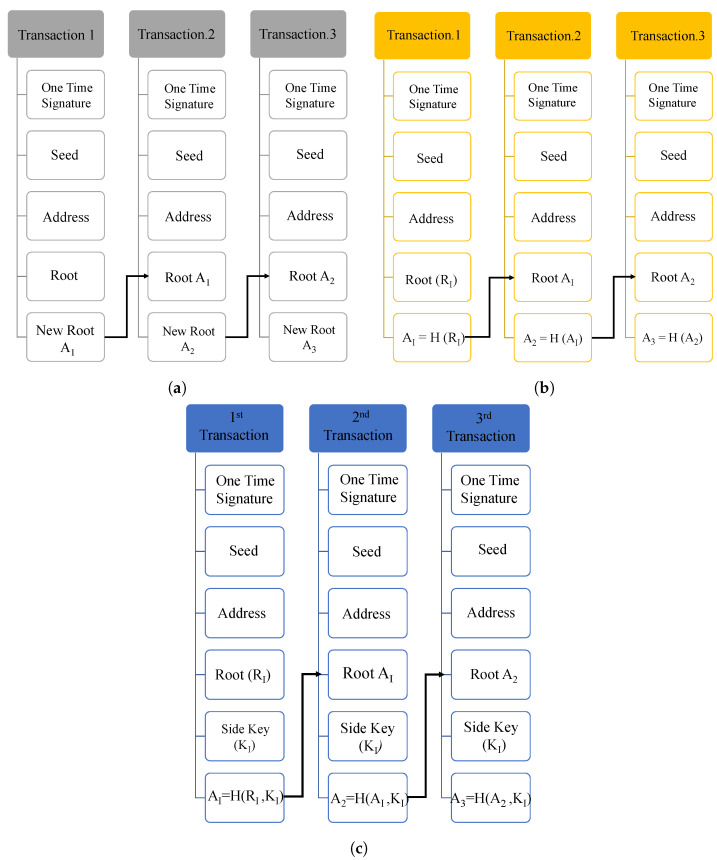
Masked authentication messaging. (**a**) Public mode; (**b**) private mode; (**c**) restricted mode.

Each channel mode has a distinct functionality and security level based on the application. Each transaction on the MAM channel has a reference to the next transaction address, which links all the transactions on that channel. However, each MAM mode has a different way of working to access the new transaction address as illustrated below: [42,43,44]. MAM works mainly in three modes: public, private, and restricted. The working flow of MAM in public, private, and restricted modes is illustrated in Figure 8.

***Public mode:*** In the public channel mode, the Merkle tree root is used as the MAM transaction address. A MAM channel with an address is generated to secure the information exchange. The address of the channel will be the root of the Merkle tree. The subsequent transaction must be submitted to the MAM channel using this fetched root, and anyone with the channel ID or address can access the channel and receive the messages.

***Private mode:*** In the private mode, the address of a MAM transaction is obtained by hashing the root of the Merkle root. For applications requiring privacy and confidentiality, as in the case of health record management, the private mode is suitable and efficient since only the subscribers with the root can decrypt the messages.

***Restricted mode:*** The restricted mode of MAM works by using a channel *authorization key* or *side key* along with the Merkle root. In this channel mode, along with the root, the side key is also hashed to obtain the transaction address on the channel. This mode provides the highest level of security for the transactions on MAM since only subscribers with an authorization key can access the transactions on the channel.

## 6. Overview of Blockchain Technology

**Figure 9:**
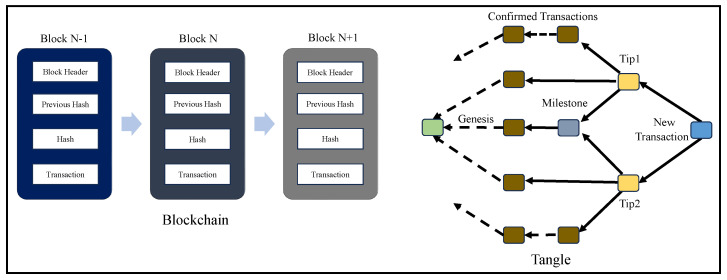
Blockchain vs. Tangle.

The success of blockchain in providing integrity and authenticity to data is not just limited to H-CPS but also in other areas of CPS, like smart transportation, Industrial IoT, and Agriculture CPS. A simple decentralized data validation and verification system provided by blockchain has made it the most alluring research area in the 21st century. Each transaction in blockchain is stored inside a block of data, which is hashed and has reference to the previous block’s hash. Miners are responsible for block validation in blockchain [11]. The validation of a block is performed through a consensus mechanism that defines rules for choosing the miners and validating the transactions. Research on blockchain consensus mechanisms has become a focus area for the research community. In all the blockchain consensus mechanisms, a miner is required to validate the transaction, and various checks and balances are in place to negate the probability of fake block generation and validation. The 51% percent attack is one of the challenges of blockchain where fake nodes could control 51% of the block addition process [19]. Blockchain technology has been perceived to be a breakthrough in realizing the potential of digital ledger technology (DiLT) for IoT-based applications. Blockchain’s robustness and features have made integration with various technologies like AI and ML an important area to work on. As various security solutions using blockchain for data have already been proposed, more emphasis is being laid on exploring the possibilities for hardware-assisted blockchain for security [12,45]. Blockchain and Tangle have varied data structures. In blockchain, the transactions are validated and added inside blocks which are aligned sequentially. Tangle is based on the Merkle tree, and it does not take much time to check whether a transaction is fake since it is a tree-based structure generation scheme [10,43]. Tangle transactions are signed using a one-time signature scheme (OTS). The Merkle tree consists of private keys as leaves which are hashed and consolidated to obtain the root address. Figure 9 presents a comparative perspective of blockchain and Tangle.

PoW, proof-of-stake (PoS), and proof-of-authentication (PoAh) are prominent consensus mechanisms. Each consensus mechanism has unique advantages and challenges that ensure a sustainable block validation process in the blockchain. Blockchain’s prime working principles are confidentiality, integrity, and authenticity. All the advanced applications such as smart cities, healthcare, agriculture, and transportation have blockchain-assisted security solutions, as they guarantee and provide integrity and immutability to data and facilitate decentralized access control. The PoW consensus mechanism involves block validation, which works based on solving a mathematical puzzle to obtain the hash value of a transaction. However, it has more computational and energy resource requirements. PoS includes a stake-based miner selection approach, which works by selecting a miner with a large amount of stake. This approach can centralize the block validation to the nodes with a higher amount of stake. For hardware-assisted IoT-based applications, PoAh presents a device authentication mechanism that verifies the integrity of IoT devices to accept the data and validate transactions in IoT applications. Blockchain has been classified into public, private, and consortium based on the number of nodes in the network. Public blockchains have many nodes, whereas private blockchains have a limited number of nodes. Public blockchain has privacy issues since the copy of each transaction is shared globally among various stakeholders in the network. A consortium blockchain is a hybrid one that has features of both public and private blockchain.

EHR management is one of the most important applications of blockchain in healthcare. EHR stores the data, provides access only to authorized individuals, and can restrict unauthorized access. Private, public, and consortium blockchain architectures achieve data confidentiality depending on the access control. Decentralized ledger technology (DeLT) is a database accessible to all trusted parties in the network to read and access the data. DLT, on the other hand, enables the trusted parties to upload and update the changes to data in the database.

## 7. PUFchain 3.0: Proposed Security-by-Design (SbD) approach for Smart Healthcare

In this section, we briefly illustrate the architectural overview of the proposed SbD approach and its working in different phases in Section 7.1. The notations used for each of the components and their associated operations are given in Table 3.

### 7.1. Design and Analysis of Proposed Framework

The proposed work explores the scope of hardware-assisted distributed ledger and blockchain for robust security in H-CPS. The proposed framework uses blockchain’s smart contracts, IOTA MAM, and PUF primitives for the security of devices and data in smart healthcare. In the proposed approach, the PUF-embedded smart sensors in the patient’s health network or BAN could securely connect to the patient’s gateway that is further connected to an edge for the secure verification of PUF keys of IoMT devices. Once the verification is successful, the edge node initiates a MAM channel creation and uses the patient’s gateway PUF key as the MAM channel side key for that hub. MAM is used to securely transfer data and upload data on Tangle. Therefore, each patient’s physiological sensor data could be shared globally among various stakeholders in the H-CPS through a PUF-based integrity-checking scheme. Blockchain in the proposed framework works on storing each patient’s PUF-generated device identities in a hub and can only be accessed by authorized stakeholders globally. This approach reduces the exposure of PUF keys of IoMT devices and reduces the need to store the PUF keys of all the devices inside a patient’s hub. MAM can work on the patient’s gateway key to securely access and upload data from these devices. Blockchain is operated by the stakeholders when a patient’s sensor hub must be accessed and the devices’ integrity must be verified.

**Figure 10:**
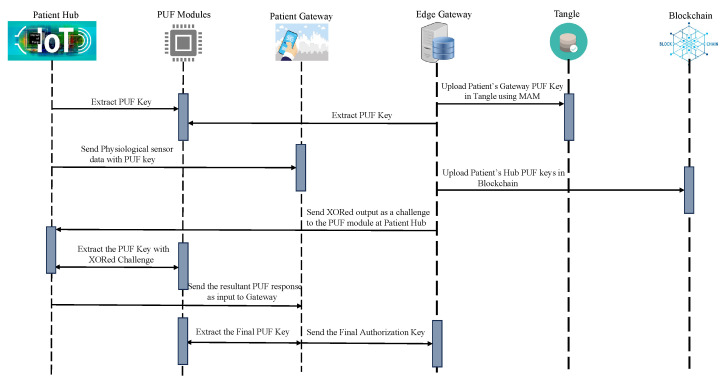
Procedural flow of PUFchain 3.0.

1. ***Patient’s sensors and gateway registration phase:*** Initially, all the smart wearable and implantable medical devices are connected to a patient’s gateway. These devices are connected to the gateway through various technologies like NFC, ZigBee, and BLE. All these devices have a PUF-embedded key as their pseudo-identity. The gateway also has a unique PUF-generated identity which acts as the address for this hub of devices. When the edge gateway receives an initiation request from the patient’s gateway, it securely verifies the gateway’s integrity by performing PUF key extraction and validation. Once the validation is successful, the Tangle transaction validation process starts. Initially, the edge gateway connects to a public IOTA node for securely interfacing with IOTA Tangle. The IOTA node then creates a MAM channel to upload and share data. In the proposed approach, the MAM channel operates in the restricted mode, which requires an authorization key for uploading and receiving data onto Tangle. The patient’s gateway transaction is securely uploaded onto the channel. Uploaded transactions could be shared among various stakeholders, who can only access in the restricted mode. The procedural flow of transaction initiation, PUF key validation, and its metric evaluation process are illustrated in Figure 10. Only after verifying the PUF reliability, uniqueness, and randomness are the PUF module keys assigned as pseudo identities to devices.

The microcontroller connected to the client broadcasts the PUF keys to the edge server (ES). Algorithms 1 and 2 illustrate the working flow of the device registration phase in PUFchain 3.0.

**Figure 11:**
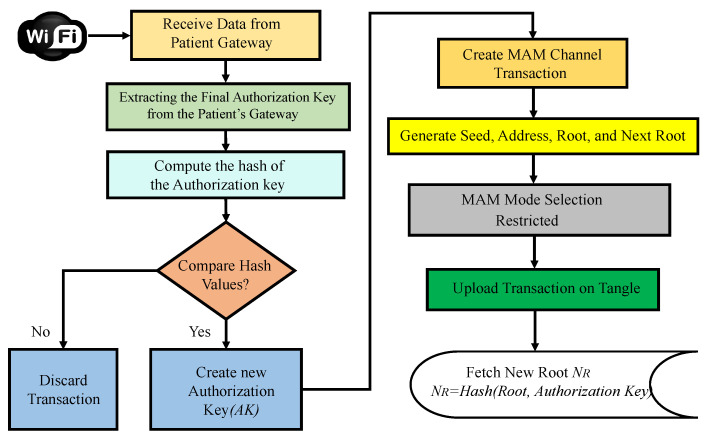
Procedural flow of MAM channel creation and transaction initiation.

**Figure 12:**
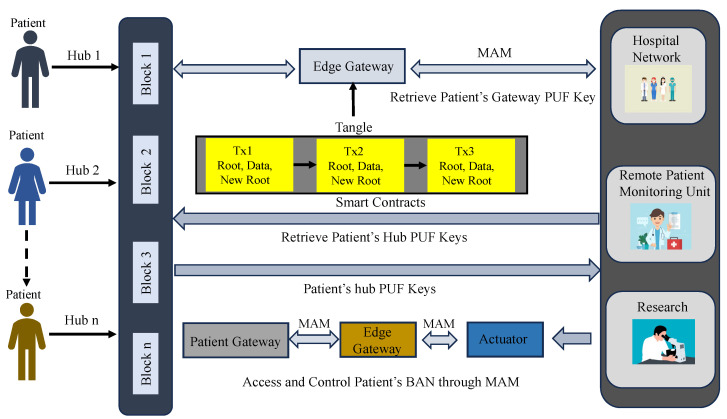
Blockchain-based access control.

2. ***Patient’s gateway access and control phase*** In MAM, while validating a transaction, a new root address is generated, which is the subsequent transaction’s hash. This is shared only with the intended recipient to successfully upload a new transaction. Using the side key, the new transaction’s root is obtained by hashing the existing transaction’s root with the side key [10,43,46]. Once the gateway’s key is verified, its details are shared on the MAM channel by creating a transaction. The recipient can be either a server at a hospital, physician, or any other healthcare provider who can access the channel to receive it only after their PUF pseudo-identity verification. Figure 11 and Algorithm 3 outline the validation and verification details. Now each administrative server at any hospital network around the world looking to access the patient’s sensitive physiological data and access the IoMT devices on patients can securely connect to the patient’s gateway hub from Tangle. A global blockchain in the cloud having all the patient’s hub PUF keys can be accessed by the corresponding hospital network or healthcare provider to obtain the individual device’s PUF key in a patient’s BAN as explained in Figure 12. The pseudo-PUF identities and challenges of all the devices are stored inside a blockchain and can be shared globally.

**Algorithm 1:** Enrolling a patient’s Body Area Network devices.

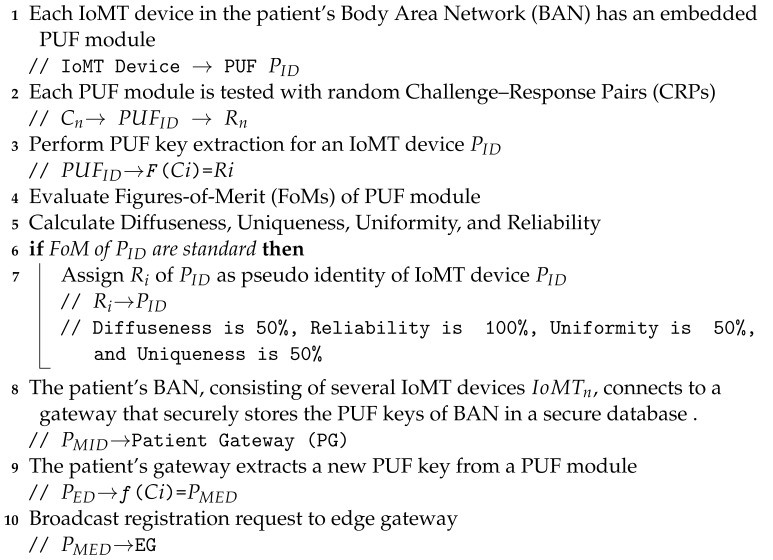



**Algorithm 2:** Patient’s gateway pseudo identity verification phase.

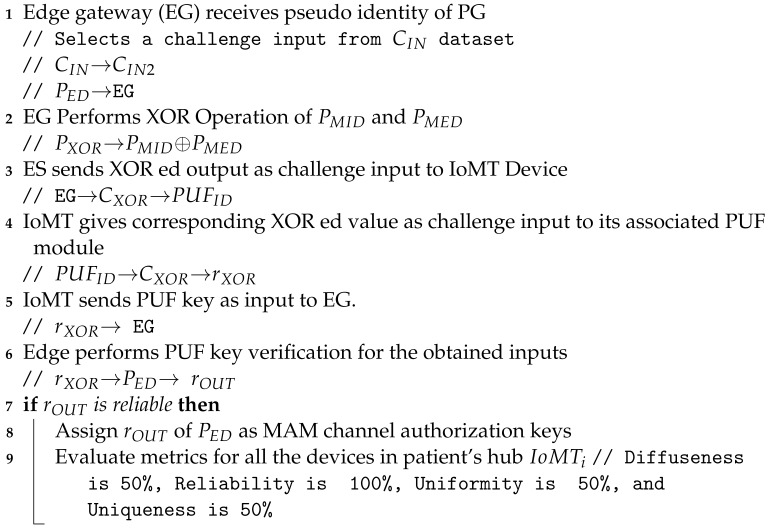



**Algorithm 3:** MAM channel and blockchain validation phase.** 1**  EG initiates MAM channel** 2**  Assign authorization key     //
MAM Channel→$A_{K}$

    //
MAM Mode →Restricted(2), Public(0) ,private(1)** 3**  Choose Restricted Mode (2) ** 4**  Upload Pseudo Identity of patient’s hub and patient’s gateway. //$P_{MID}$→     Streams v0 (Channel)** 5**  Choose patient’s gateway key as the channel side key      //
$P_{MED}$→$A_{K}$** 6**  Fetch Next root      //
MAM Channel →New Root($N_{R}$)** 7**  Perform hash on side key and root     //
$A_{M}$→H($A_{K}$,$R_{K}$) ** 8**  Broadcast new fetched root and new side key $A_{M}$     // --------EG initiates blockchain transaction-------** 9**  EG initiates a smart contract with different roles: doctor, patient **10** EG uploads the patient’s hub PUF data set     //
-----$IoMT_{n}$-----    
// 
$IoMT_{i1}$ →H($C_{i1}$,$R_{i1}$)    
//
 
$IoMT_{i2}$ →H($C_{i2}$,$R_{i2}$)    
//
 
$IoMT_{i3}$ →H($C_{i3}$,$R_{i3}$)    
//
 
|    
//
 
|    
// 
$IoMT_{in}$ →H($C_{in}$,$R_{in}$)**11** Deploy smart contract **12** Obtain mined and validated block **13** Broadcast validated block globally to various stakeholders

**Figure 13:**
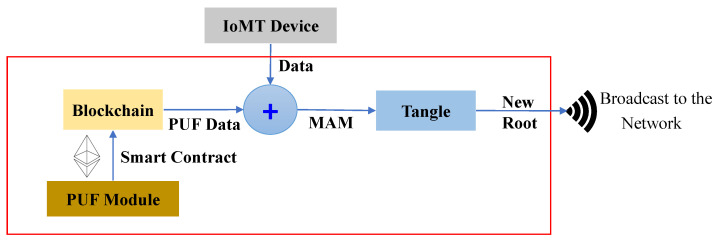
Working flow of PUFchain 3.0.

The patient’s gateway key is verified by the edge gateway, which then initiates a new transaction on the IOTA MAM channel. After uploading the transaction, it is shared on the channel, and the intended receiver can access the data in restricted mode. The working and procedural flow of the uploading transaction on MAM channel creation and its validation inside a node in the proposed PUFchain 3.0 is presented in Figure 13.

***Step 1:***  The IoMT device’s integrity is verified by performing PUF key extraction from a set of challenges on the device’s PUFs.
***Step 2:***  The challenge inputs ($C_{i}$, $C_{k}$) are tested on the PUF modules at both the gateway’s and device’s PUF modules in the hub.
***Step 3:***  The obtained keys are evaluated by checking the reliability, randomness, Hamming distance, and other metrics.
***Step 4:***  XOR operation is performed on the obtained PUF keys ($P_{MID}$, $P_{MED}$). The XOR output $C_{XOR}$ is sent as a challenge input to PUF at IoMT.
***Step 5:***  The obtained $r_{XOR}$ key is again tested as input to the PUF module at the gateway.
***Step 6:***  Finally, the obtained key from the gateway is hashed and compared during the verification process by following all the above steps. The obtained final key $r_{OUT}$ is hashed. The obtained hash value $H_{A}$ is compared with the initially obtained hash $H_{D}$ during registration.
***Step 7:***  Once the device authentication is considered successful by the edge gateway, it then creates a MAM channel to upload the transaction, fetch the address, and broadcast it to the authenticated client to upload its data.
***Step 8:***  The working mode of MAM is chosen as the restricted mode (2). An authorization or side key $A_{K}$ is defined to access the channel in restricted mode.
***Step 9:***  The authorization key $A_{K}$ for the MAM channel in the proposed security protocol is the patient’s gateway pseudo identity $r_{OUT}$, which is required to store, share, and access data on IOTA Tangle.
***Step 10:***  Once the new root is fetched, an access link is obtained and broadcasted to all the working nodes in H-CPS to access the transaction data from Tangle.
***Step 11:***  Finally, the root of the transaction $R_{K}$ and $A_{K}$ of the MAM channel are hashed to fetch the address ($A_{M}$) of the new transaction. The new side key is $r_{OUT}$ of the patient’s BAN gateway.
***Step 12:***  The subsequent transaction address ($A_{M}$) is used for data transfer from all the other stakeholders to the intended recipient on the MAM channel, thereby creating a secure communication channel for the patient–doctor interface in H-CPS.

### 7.2. Assumptions

The proposed experimental validation is based on the following assumption.

- All the IoMT devices have embedded PUF.
- A secure network communication exists between the IoMT node and the patient’s and edge gateways during the enrollment and verification process.
- All the IoMT devices have a secure interface with the patient’s gateway using BLE, ZigBee, or other technologies.
- The edge gateway has a running blockchain instance locally.

## 8. Experimental Results

**Figure 14:**
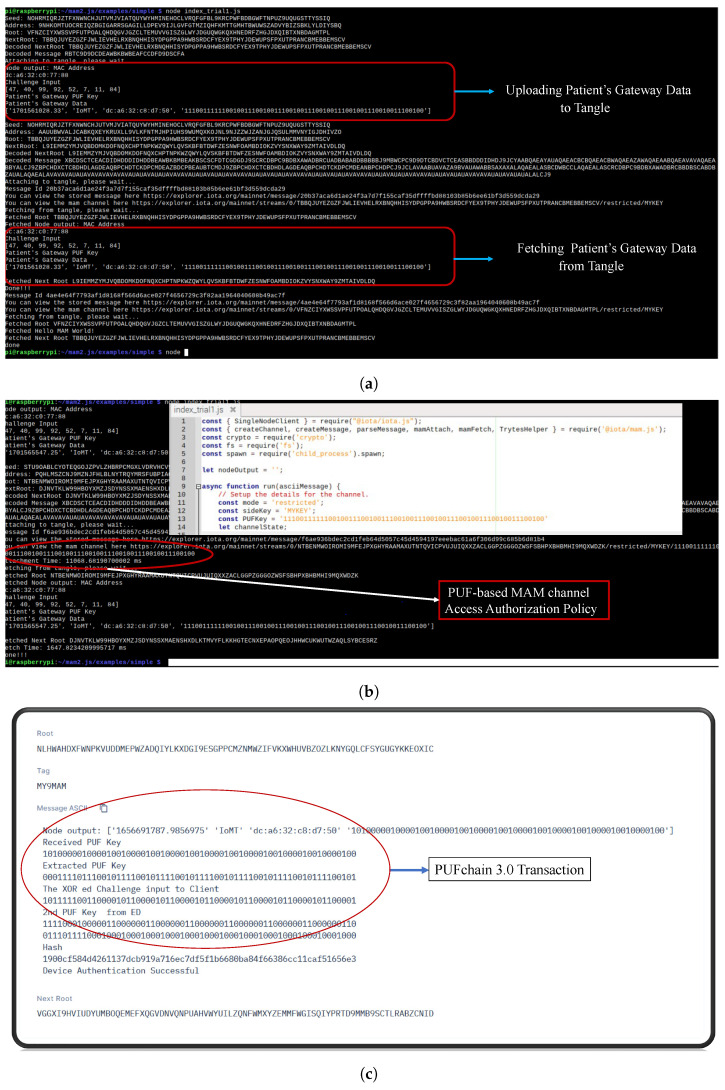
IOTA Tangle transaction validation. (**a**) Validating PUF key and creating MAM channel; (**b**) PUF-Based MAM channel access authorization policy; (**c**) fetching transaction from IOTA explorer.

For experimental evaluation, all smart health devices inside the patient’s BAN are interfaced with the patient’s gateway, and all the data processing can be performed at the edge gateway. Two FPGA boards are used for PUF module deployment on the patient and edge gateway side. The patient’s gateway has an Arbiter PUF generated key, and the edge has an XOR PUF Key as unique identities. Arbiter PUF can generate many keys for patients’ BAN smart health devices. The proposed methodology was written in JavaScript to publish and fetch transactions on Tangle. We used the Chrysalis public IOTA node to access and upload transactions on the MAM channel. MAM channel in “restricted mode” was considered for the proposed approach to ensure higher security. The whole methodology was evaluated on the IOTA Main net on Streams v0 Channel [47,48]. The hardware and software specifications of the experimental validation in this work are given in Table 4. The time taken to upload a transaction on Tangle is the total time to generate *Tip*, validate the transaction using PoW, and generate a MAM channel and corresponding transaction metrics—*seed, address, root*. Our experimental evaluation has shown that the overall time to perform transaction validation in the proposed work is comparatively faster than that of block addition in PoW, which is approximately 10 min [19]. The transaction evaluation and validation results are presented in Figure 14.

**Figure 15:**
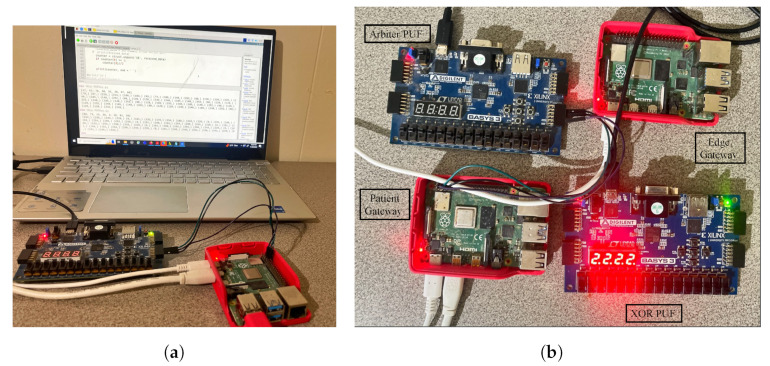
PUFchain 3.0 Experimental Setup. (**a**) Extracting PUF keys; (**b**) prototype.

A Ganache local test net blockchain was set up and connected to a MetaMask account for gas cost estimation and analysis. A smart contract was deployed to securely store the generated PUF Challenge–Response Pair (CRP) dataset inside the blockchain. The Ganache blockchain was configured on an Intel i7 2.8 GHz processor with 16 GB RAM (Intel, Santa Clara, CA, USA). Xilinx (AMD, Satnta Clara, CA, UUSA) FPGAs were used for evaluating the Arbiter and XOR PUF modules for PUF key extraction as shown in Figure 15. The FPGA boards used for evaluation are Xilinx Artix-7 Basys 3 (xc7a35tcpg236-1). Xilinx Vivado was used to test the PUF design, and the PUF logic was programmed onto the FPGA board at a baud rate of 9600 bits using a Universal asynchronous receiver and transmitter (UART). The 64-bit instances of Arbiter and XOR PUF elements were generated to create 64-bit PUF keys for each one of the modules. Table 5 presents the Arbiter and XOR PUF evaluation results.

Single board computers were used as edge nodes for distributed data processing from the IoMT devices. Raspberry Pi 4 2.0 GB boards were used as the edge and patient’s gateway in the proposed system. These devices act as local nodes to perform device integrity verification and for creating MAM channel and uploading transactions on Tangle. The edge gateway’s power consumption was evaluated using an energy meter, which showed power consumption in the range of (2.7–3.4) watts, which is approximately the average consumption range of a pi. The PUF keys of each of the devices were initially verified before creating a new MAM channel and uploading the transaction onto Tangle.

**Figure 16:**
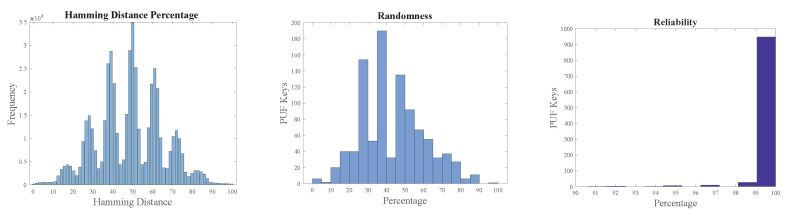
Arbiter PUF metrics.

**Figure 17:**
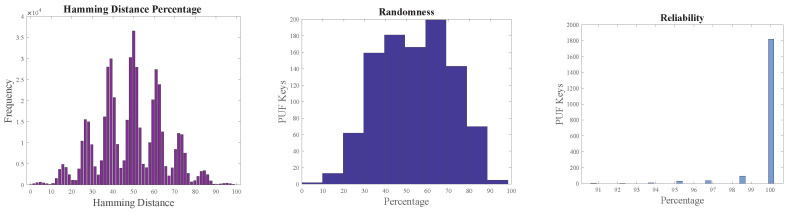
XOR PUF metrics.

The overall intra-Hamming distance of PUF keys from Arbiter and XOR PUF modules was approximately 50%. The metrics of PUF modules are presented in Figure 16 and Figure 17. Reliability was approximately 100% when the two PUF modules were tested with 1000 PUF keys four times at different instances of time and at varying temperatures.

### 8.1. Why Restricted Mode of MAM for PUFchain 3.0?

MAM as introduced in Section 5, works in public, private, and restricted modes. However, the proposed approach works on MAM in the restricted mode. This is due to the requirement for device and data integrity from smart electronic devices. Restricted mode ensures the utmost level of security and works by generating a transaction address by hashing the hash of the root and an authorization side key. This work aims to leverage this property by using the PUF key of a device as its authorization key to access the channel and upload data to Tangle. In the proposed solution, doctors and medical professionals can access the channel securely and obtain access to the data from Tangle. This can ensure the integrity and authenticity of data, as the data can only be uploaded onto Tangle after successfully validating the PUF keys of respective Medtronic devices.

The overall time to perform device authentication in PUFchain 3.0 is between 2.7 and 3.6 s. Once the device authentication is complete, the average time to upload the transaction onto Tangle Main net is 28 s, while the mean time to fetch the transaction is approximately 1–2 s. The tabulated results of PUFchain 3.0 are given in Table 6. The transaction upload time includes the time taken to perform seed, address, root, and other Tangle transaction metrics. Also, it includes the waiting time for the IOTA public node to attach the transaction to Tangle and the time taken to perform PoW to validate the unconfirmed transactions on Tangle.

### 8.2. Block Creation and Validation

**Figure 18:**
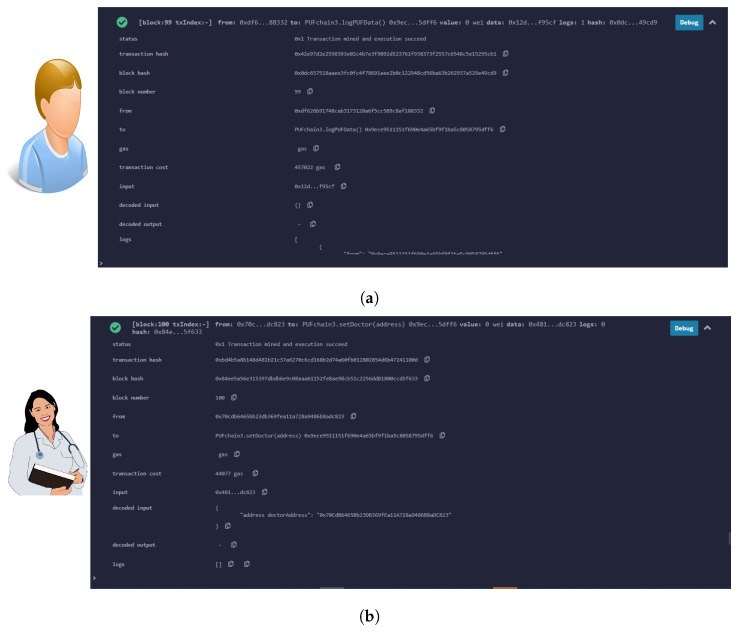
Smart contract deployment and role assignment. (**a**) Assigning patient’s role and logging PUF data; (**b**) assigning doctor’s role and retrieving PUF keys of patient’s BAN.

**Figure 19:**
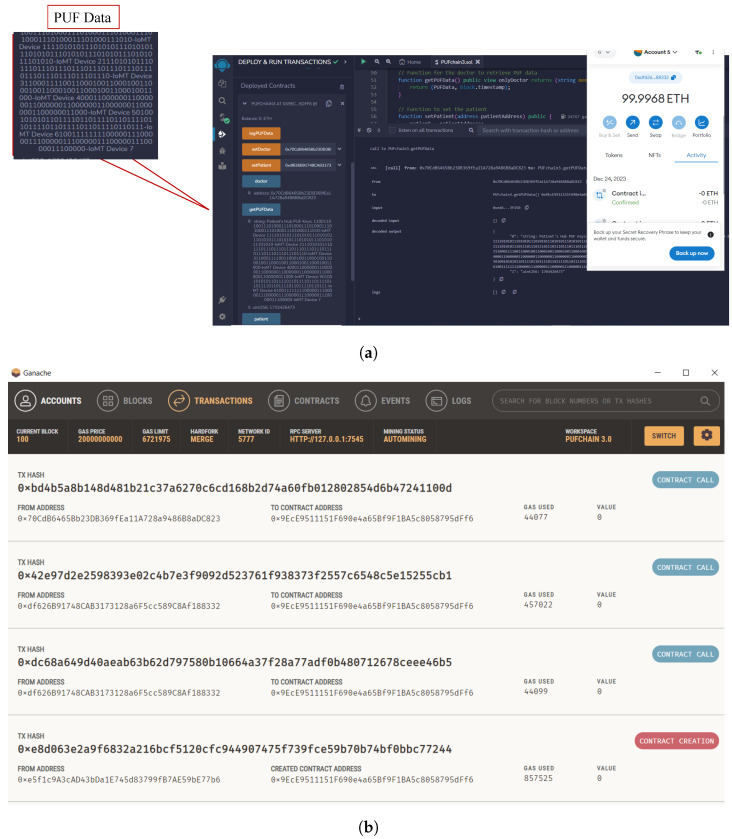
Formal verification of PUFchain 3.0. (**a**) Transaction details of PUFchain 3.0 on Remix IDE; (**b**) Validated PUFchain 3.0 transactions on Ganache.

A smart contract was deployed on Remix Ethereum IDE connected to a MetaMask wallet. Ganache blockchain running on a local host was connected to MetaMask, and one of the ten accounts was selected and connected to the MetaMask wallet. A simple smart contract to store the Patient’s PUF data set consisting of PUF keys was executed and deployed onto the Ganache test network [49]. The block creation, hashing, and transaction validation results are presented in Figure 18 and Figure 19. Our prototype system worked on the local Ganache test net using network ID 5777 and smart contract address “0xe5f1c9A3cAD43bDa1E74 5d83799fB7AE59bE77b6”. Two accounts were assigned, one for healthcare professionals with contract address “0x70CdB6465Bb23D B369 fEa11A728a9486B8aDC823”, and one for the patient using the address “0xdf626B91748C AB3173 128a6F5cc 589C8Af18 8332”. The “logPUFData” function is initiated from the patient’s side which securely logs the PUF keys of IoMT devices. The doctor or health professional initiates the “PUFData” function to securely retrieve the PUF keys of IoMT in the patient’s BAN. The smart contract validation results of the proposed framework are given in Table 7.

## 9. Discussion and Conclusions

### 9.1. Principal Findings

This work explored the potential of hardware-assisted distributed ledger technology-based security solutions in smart healthcare. We proposed a cybersecurity solution for H-CPS by integrating PUF, IOTA Tangle, and blockchain. Tangle, being a distributed lightweight ledger, offers great potential in smart healthcare, as it is a minerless, feeless primitive while offering robust security as blockchain. We experimentally demonstrated a security solution that uses blockchain for securely storing the PUF keys of each of the IoMT devices in a patient’s BAN. The patient’s gateway, having a unique pseudo identity from the PUF, can communicate on MAM for sharing physiological sensor data globally.

This work demonstrated and evaluated two PUF modules: Arbiter and machine learning attack-resistant XOR Arbiter PUF. One thousand PUF keys were extracted from these PUFs for five instances, showing promising results with a reliability of approximately 100%. Our analysis of related works shows that most of these works do not focus on PUF metrics and hardware-assisted access control to the distributed ledger. Our work presents a hardware secure access control policy to DLT with the effective evaluation of PUF metrics to facilitate an attack-resistant security framework. Table 8 illustrates the comparative analysis of this work with related works.

Our analysis further proves that even though Tangle MAM has been proposed in various works, it has not been integrated with hardware primitives as a comprehensive cybersecurity solution. To the best of our knowledge, this is the first novel integration of PUF, blockchain, and Tangle for simultaneous device and data security in smart healthcare or other areas in IoT-based applications.

Our security analysis shows that eavesdroppers cannot intercept the communication and PUF keys of the patient’s gateway shared on the MAM channel since the restricted mode channel ensures secure access using the patient’s gateway PUF key. Also, consecutive transactions can be uploaded onto the channel only by sharing the obtained new root address and channel side key with the trusted authorized entities in the system. As a result, the proposed approach can withstand eavesdropping attacks. Additionally, our analysis shows that the message attachment times in restricted MAM mode are comparatively faster in this work as compared to [9], even though the public IOTA node’s processing time may vary subject to network traffic. Also, in this work, the PUF keys of IoMT inside BAN are not shared on the MAM channel but are securely stored in the blockchain, which can be accessed by authorized entities through smart contracts, thereby reducing the exposure of smart health devices’ unique PUF-generated identities. Furthermore, the Arbiter and XOR PUF modules have shown better randomness and reliability in this work as compared to the hybrid oscillator arbiter PUF in [19]. Achieving approximately 100% reliability substantiates the potential of PUF-based security for IoMT devices.

### 9.2. Limitations and Challenges

Using public IOTA nodes for validation, publishing, and fetching data on Tangle could delay and increase transaction validation and publishing times. Using smart contract-based validation can increase energy consumption and require computational resources. Other challenges also exist with the integration of PUF, Tangle, and blockchain, such as latency in transaction validation, network security issues, and blockchain smart contract validation cost or gas fees. Even though our approach works on the Ganache test net blockchain, the actual deployment on the main net could incur gas costs. For the deployment of transactions on Tangle, MAM has been updated to a new protocol called IOTA streams [50] which is still under the development stage. Furthermore, integrating PUF for hardware security is a challenging process, as the reliability of PUF can be impacted due to the aging of the device and its response to environmental factors. Also, various trade-offs involved in the performance of PUF-embedded devices must be considered such as energy consumption, area, and speed while deploying PUF on smart health devices.

### 9.3. Conclusions and Future Research Directions

Hardware-assisted security solutions using blockchain and distributed ledger have great potential for cybersecurity in smart healthcare. The privacy and integrity of patients’ sensitive medical data are pivotal in the rapidly evolving remote healthcare monitoring systems facilitated through IoMT devices. Integrating a decentralized hardware–software SbD approach which emphasizes integrating security based on the design of an electronic system in H-CPS is the motivation for this work. The proposed work successfully integrates PUF, blockchain, and IOTA Tangle as a scalable decentralized security primitive that provides sustainable and simultaneous security in H-CPS. The proposed architecture aims to leverage the scope of blockchain technology to store the patient’s BAN PUF keys to avoid the possibility of exposure and adversarial access to these keys. Using Tangle in this work securely facilitates identity-driven access control and data sharing among various stakeholders in H-CPS for processing patients’ critical health data in real time. Furthermore, PUF enhances and focuses on security at the end device in the BAN hub. The possible integration of these three could further facilitate a secure interface between doctor and patient in advanced remote healthcare monitoring systems like telemedicine and e-health.

This work could be extended for sustainable security in autonomous vehicles by embedding PUF inside electronic control units, and it has a blockchain-supported functionality for data security as well. The proposed PUFchain 3.0 could be extended further to other areas of IoT-based applications, particularly in the areas of supply chain management and product tracking in electronics. This includes attaching a PUF-generated cryptographic identity to each product in the supply chain and tracking its movement securely using blockchain. The integration of these primitives for IC supply chain management and Industry 4.0 can also be a direction for future research.

## Figures and Tables

**Table 1:** Comparison of PUFchain variants.

Research Work	Features	Security Approach
PUFchain [19]	The PUF-generated keys are securely stored inside the blockchain for securely binding the identity of each device inside the blockchain. The PUF keys stored inside the blockchain can be retrieved securely for advanced applications requiring security for IoT devices.	Proof-of-PUF-Enabled-Authentication (PoP)–PUF based blockchain.
PUFchain 2.0 [11]	In PUFchain 2.0, for security and privacy in IoMT, a novel PUF-based blockchain solution for IoMT device and data security that has a two-level authentication mechanism is proposed. This approach has MAC address-based verification as an initial stage followed by the PUF key verification stage.	PUF-based blockchain with MAC address verification
PUFchain 3.0 [20]	For security and privacy in smart healthcare, all IoMT devices and their data are secured through a PUF-assisted distributed ledger. This approach has PUF, blockchain, and Tangle for simultaneous device and data security in H-CPS.	PUF-based distributed ledger using MAM and smart contracts

**Table 3:** Notations.

Notation	Description
$P_{MID}$	Pseudo identity of IoMT device
$P_{ID}$	PUF module at device
$C_{i}$	Challenge to IoMT device PUF
$C_{k}$	Challenge to gateway’s PUF
$R_{i}$	Response to $C_{i}$
$IoMT_{i}$	Patient’s hub
$P_{MED}$	Pseudo identity of patient gateway
$P_{ED}$	PUF module at gateway
$PUF_{n}$	PUF modules of all IoMT devices in patient’s hub
$C_{n}$	Random challenges inputs
$R_{n}$	Response
$C_{i}$	Challenge input to IoMT device $IoMT_{i}$ in hub
$R_{i}$	Extracted response from $PUF_{I}$ of $IoMT_{i}$ in the hub
$R_{p}$	Response output from patient’s gateway PUF module $P_{ED}$
$C_{XOR}$	XORed output of $R_{i}$ and $R_{p}$
$r_{XOR}$	Response output OF XORed input
$r_{OUT}$	Final key from PUF module $P_{ED}$
⊕	XOR
$A_{K}$	Side key
$R_{K}$	Merkle root
*H*	SHA-256 hash function
$H_{D}$	Hash value during registration
$H_{A}$	Hash value during authentication
$A_{M}$	Fetched new transaction root

**Table 4:** System specifications.

Parameters	Results
Application	Smart Healthcare
DLT	IOTA Tangle and Blockchain
PUF Module	Arbiter and XOR PUF
Programming	JavaScript, Verilog, Python, Solidity
IOTA Network	Main net
Tangle Communication Protocol	MAM
IOTA Node	Chrysalis
Working Mode	Restricted
MAM channel	streams v0
FPGA	Artix-7, Basys-3 (xc7a35tcpg236-1)
Block Validation	Solidity 0.8.18
Blockchain network	Ganache

**Table 5:** PUF Evaluation results.

PUF Metrics	Results
PUF Key Extraction time	78 ms
XOR PUF Reliability	99.72%
Overall Hamming Distance of XOR PUF	48.66%
Overall Hamming Distance of Arbiter PUF	48.53%
Arbiter PUF Reliability	99.73%
Number of PUF keys	1000
Number of Instances	64
Total On-Chip Power	0.081 Watts
Device Authentication Time	3.66 s

**Table 6:** Tangle transaction evaluation results: analysis of 10 sample transactions.

Message ID	Attachment Time (s)	Fetch Time (s)	Root
9d9646d0d0536ee 9aede181660ab799 247b58548fe09 107e421643ae3c2581b3	13.8	1.38	KJAMAHXDTWOSOJAJ99UMX XRBBKHHUD NDHJVLTBNRQD UFSRQEQZDNYKTS BNGKUTUPYXYC STXLLZXSDP9KR
f2a2766970d6044 705af5d14fce0f5e0 e844b6a460bd 1960caf82148c0aa3600	26.6	1.66	HEXQBCPQSZYYJQXUMB UYKHRSNUOJNUU CPZFNAJLZDSZEUUAE RLLSPLKTBPVEHHECU TKDETPPXKXVYTXAG
2ac926abc3eeb3 11eaf8356945358b ced6e3836ef7e43d 84f517d756a551970d	23.0	1.33	ZCEOYFYQB MFXMAWMDHTUZ ZNJMJGA SEVBGBMOU LNHKSWZ OCAER9 KGXOEECLDWRJM CJJEVGRBAAYKINTSTM
daee1db6f01b59 4f07efaf1e04e 012e01fd ce53e714a83a 0414abb5256064ca5	22.5	1.67	E9ESRZ9B SXIXON9URUACLVJ BLHHNKUFGRI9D9 BQJUCAKWI9YQVTVT DAQCIWLQPSMXWUNCT QPTSBIUVUYF
152518578c56268af d2380bcedd64a 37379b7e200d20a dbbab9c71866567eee1	36.1	1.90	GNUJKSBQOGW JZTLXDHDSUFAFVTWH POQXXL9AVOAYZ VVU9YP LRSAKWNGTQ9W TGEURIP STYBOJLMCXGBTIW
b4c291bbc8b867d 7b912ab9a2cad 3e6d8bb8b 15fa022b3 db7cb14cf88f8c9775	20.5	1.52	OBSFYFONDRKIXRDWWB9T BQZYOMVOYK USLGAXYBS9VD MTMNZCXYYOVQX UU9OWUHWR DRHLHMRU KNHPTBMEH
3877bf6821b5df c36823ce a6eee1a e23b5b61 73c4e080 0dbd58 26516b8 5bbca8	2.16	1.61	GXNHDCAVIAUAIDPESPJ BBBYLH9PSIK9FJHMG ALYLAJAQUP ZOV9KIBNFXMBX HJAASZ ZATLE UQQGHEYO9IV
bee8195b378 2a51443 afb2087d91 eb5743 e31dcdb15f42 32d6ac8e932d 7d3513	7.80	1.51	SKGKMHKG9ZNIN JOXMDIONLULRFBZOQFDLQ TAIKUAOIQNMNQT DSYVS9SZKDTAB CYRVVOEARA9UWDFWVPBE
dde4579afe5 e10bb6a7 a5e0fb8b461 f62d752023e 38769f001f6 e7e5ea95e3a1	13.0	1.44	9GIY9J9UDCN CSYUKZKXBRSJQDZBIU9G HOBGNEBBHQ EPSZYKNCH9LSOBID9 BLPW9TSTNDLHWX JAXNVVASE
0dc5cfe486b1 ce772d8459b a5f95bd2836 2d8b69cfa 843fc4fc 47caa7d39c3a7	11.6	1.77	OMOTIFWLJ9DNRJ QBCGBIBMEMAMYKL FKCFMZOLSC C9WOWVWEO ICYFQDIY9UW HEIADXGMFATZU NJRLCTITK

**Table 7:** Smart contract deployment details.

Smart Contract Lifecycle	Transaction Hash	Block Hash	Gas Fees
Contract deployment	0xe8d063e2a9f6 832a 216bcf5120c fc944907475f739f ce 59b70b74bf0bbc77244	0x78d0ef9a76714407c3 1d777b40f8ce0da579ba9181 729cb753b9fe19d26ce73f	0.02600838 ETH
Patient’s account initiation	0xdc68a649d40aeab63b6 2d797580b10664a37f2 8a77adf0b480712678ceee46b5	0x7488a604b74b7d9e7 404fac9705108c6ae25f530 d3f39aee97b93cdc2acec58f	0.00132949 ETH
PUF data storage	0x42e97d2e2598393e02c4b7 e3f9092d523761f938373 f2557c6548c5e15255cb1	0x0dc657518aaee3fc0fc 4f78691aee2b0c1229 48cd56ba 63b262937a529e49cd9	0.01637317 ETH
Doctor’s account validation	0xbd4b5a8b148d481b21c 37a6270c6cd168b2d74a60 fb012802854d6b47241100d	0x84ee5a56e315397dbdb6e 9c08aaa61152fe8ae98 cb51c2256dd81800ccd5f633	0.00169751 ETH

**Table 8:** Security analysis of PUFchain 3.0 in comparison with related works.

Research Works	System	Security Primitives	Hardware Assisted	Scalable	Hardware Efficient	Computationally Efficient
Wang et al., 2022 [30]	PUF and Fuzzy extractor-enabled blockchain	3	Yes	Yes	No	Yes
Chaudhary et al., 2021 [22]	PUF-based Smart Contracts	2	Yes	Yes	No	Yes
Satra et al., 2023 [14]	ML-assisted PUF	1	Yes	No	Yes	-
Al-Joboury et al., 2021 [23]	DAG Blockchain	2	No	Yes	-	No
Fotopoulos et al., 2020 [28]	Blockchain-assisted SSI	1	No	Yes	-	No
Zheng et al., 2023 [9]	IOTA MAM	1	No	Yes	-	Yes
PUFchain 3.0 [20]	Blockchain-enabled PUF for Tangle’s MAM	3	Yes	Yes	Yes	Yes

## Data Availability

All the data generated or analyzed during this study are included in the published article. Any data related to this study can be provided with reasonable request.
